# Papain-cetylpyridinium chloride and pepsin-cetylpyridinium chloride; two novel, highly sensitive, concentration, digestion and decontamination techniques for culturing mycobacteria from clinically suspected pulmonary tuberculosis cases

**DOI:** 10.1371/journal.pone.0236700

**Published:** 2020-08-04

**Authors:** Pottathil Shinu, Varsha A. Singh, Anroop Nair, Katharigatta N. Venugopala, Sabah H. Akrawi

**Affiliations:** 1 Department of Biomedical Sciences, College of Clinical Pharmacy, King Faisal University, Al Ahsa, Saudi Arabia; 2 Department of Microbiology, M.M.I.M.S.R., M.M. Deemed to be University, Ambala, India; 3 Department of Pharmaceutical Sciences, College of Clinical Pharmacy, King Faisal University, Al Ahsa, Saudi Arabia; 4 Department of Biotechnology and Food Technology, Durban University of Technology, Durban, South Africa; The University of Georgia, UNITED STATES

## Abstract

Mycobacterial culture remains the gold standard for the diagnosis of tuberculosis. However, an appropriate digestion and decontamination method (DDM) is essential for the effective recovery of tubercle bacilli in culture. Therefore, the current study was designed to compare the performance of papain-cetylpyridinium chloride [papain-CPC] and pepsin-cetylpyridinium chloride [pepsin-CPC] DDMs against N-acetyl L-Cysteine-sodium hydroxide (NALC-NaOH) DDM for recovery of mycobacteria from clinically suspected pulmonary tuberculosis cases. To evaluate papain-CPC, pepsin-CPC and NALC-NaOH DDMs, sputum samples (N = 1381) were cultured on Löwenstein-Jensen medium and the results were compared. The papain-CPC DDM showed sensitivity, specificity, positive predictive value, and negative predictive value of 100%, 93.27%, 71.7%, and 100%, respectively as compared to NALC-NaOH DDM. Similarly, pepsin-CPC DDM demonstrated sensitivity, specificity, positive predictive value and negative predictive value of 98.94%, 94.7%, 76.11%, and 99.81%, respectively. In summary, both papain-CPC and pepsin-CPC DDMs are highly sensitive and specific techniques for recovery of mycobacteria as compared to NALC-NaOH DDM. However, when the overall performances of all DDMs compared, papain-CPC DDM isolated increased number of mycobacterial isolates with comparatively higher numbers of colonies on LJ media than both pepsin-CPC and NALC-NaOH DDMs, indicating its potential to replace the NALC-NaOH DDM for recovery of mycobacteria from sputum samples.

## Introduction

Culture is considered to be the gold standard for diagnosis of tuberculosis (TB) [1]. However, an appropriate digestion and decontamination method (DDM) is essential for the effective recovery of *Mycobacterium tuberculosis* (MTB) in culture. In the past years, various DDMs have been established to isolate MTB from clinical specimens [2–9]. However, paucity of research data and operational challenges, more specifically; availability of chemicals, cost-effectiveness of the method and lack of standardization restricted the implementation of these DDMs in laboratory settings. This necessitates the importance of further research for the development of sensitive, technically simple, digestion and decontamination techniques for the effective recovery of MTB from clinical specimens.

The current study investigated two novel, cost effective, highly sensitive, concentration, digestion and decontamination techniques, namely papain-cetylpyridinium chloride (papain-CPC) and pepsin-cetylpyridinium chloride (pepsin-CPC) DDMs. In the papain-CPC DDM, papain functions as a mucolytic agent and the CPC as disinfectant. Papain is a cysteine protease enzyme of the peptidase C1 family, which is extracted from *Carica papaya*. This cysteine protease enzyme consists of a single polypeptide chain with three disulfide bridges and a sulfhydryl group, which is responsible for the enzymatic activity of papain [10]. Papain is biologically active at an optimal pH of 6–8 and digests most protein substrates more extensively than other pancreatic proteases. In addition, papain is soluble in water at 10 mg/ml and commonly used in cell isolation procedures where other proteases are usually destructive [11]. However, CPC is a quaternary ammonium compound that has bactericidal activity particularly against normal flora present in the upper respiratory tract secretions [2, 7]. This enzyme-bactericidal combination, when mixed with sputum samples enhances both digestion and decontamination of the sputum. Similarly, pepsin-CPC DDM uses pepsin for mucolysis and CPC as decontaminating agent. Chemically, pepsin is an aspartic endopeptidase secreted as pepsinogen (a proenzyme) by chief cells in the stomach of humans and animals. This pepsinogen gets converted into pepsin (when it mixes with hydrochloric acid in the gastric juice) that causes hydrolysis of many proteins and peptides [12]. This hydrolysis activity of pepsin was utilized in the pepsin-CPC DDM of the current study. Considering the above details, the present study was designed to evaluate and compare new DDMs namely, papain-CPC and pepsin-CPC for isolation of mycobacteria from sputum specimens obtained from clinically suspected pulmonary tuberculosis (PTB) patients. The present study evaluated the performance of papain-CPC and pepsin-CPC DDMs with N-acetyl-L-cysteine-sodium hydroxide (NALC-NaOH) DDM (reference method) for recovery of tubercle bacilli from clinically suspected PTB cases.

## Materials and methods

The study was carried out in the Department of Microbiology (between June 2015 and May 2017), of M. M. Institute of Medical Sciences and Research, Ambala (India), a tertiary care hospital that also operates as a peripheral Centre for the Revised National Tuberculosis Control Program (RNTCP). Two sputum samples (spot sample at hospital and morning sample at home) were collected from consecutive patients (>18 years old); having productive cough for more than two weeks or longer or from those patients as recommended by the trained physician. No patients, who received anti tubercular treatment in previous six months, were recruited for the study. However, patients, who received antibiotic treatment for respiratory tract infections other than TB, were included in the current study. Immediately after specimen collection, sputum samples were transported to the microbiology laboratory and stored over night at 4–8°C. After obtaining the morning sputum sample, smears were prepared from each specimen, stained by Auramine O staining method and examined using Light Emitting Diode Fluorescent Microscope (LED FM, magnification, X 400) (Zeiss primo star i LED Gottingen, Germany). All the direct LED FM reports were dispatched to patients as the sputum samples were collected for routine direct Auramine ‘O’ staining. Direct sputum smear examination was used for presumptive diagnosis of clinically suspected PTB cases. After issuing the reports, both the sputum samples (spot specimen at hospital and morning specimen at home) were mixed. These mixed sputum specimens were homogenized by vortexing (3 min) to assure the homogeneous distribution of tubercle bacilli. This homogenized sputum sample was segregated into four aliquots, the first 0.5 ml was used for Auramine ‘O’ smears, the other three aliquots (2–3 ml each) were transferred to three separate 14 ml BD Falcon centrifugation tubes (BD Biosciences, USA).

### Laboratory methods

#### Direct smear

The homogenized sputum specimens were used for preparing direct Auramine ‘O’ smears. All slides were examined using a LED FM by an experienced laboratory technician and interpreted as per the standard operating procedures published by Revised National Tuberculosis Control Program (RNTCP) [13]. All the LED FM smear positive samples were also confirmed with Ziehl–Neelsen staining. Further, 10% of randomly selected negative slides and all the positive slides were checked by the study supervisor to validate the results.

#### NALC-NaOH DDM

The NALC-NaOH DDM was performed using 2–3 ml of sputum in 14 ml BD Falcon tube as suggested by Kent & Kubica with few modifications (final NaOH concentration 1%) [14]. Briefly, 4% NaOH solution was mixed with an equal quantity of sodium citrate solution (2.9%) to prepare the working solution. Before the use of the decontamination solution, NALC powder was added to yield a final concentration of 0.5%. After the DDM procedure, processed sputum (0.2 ml) was inoculated on two slants of Löwenstein-Jensen (LJ) medium (Himedia, Mumbai, India) [one LJ slant with glycerol and the other slant with sodium pyruvate]. These slants were further incubated at 37°C aerobically and culture was monitored for growth of MTB weekly once for eight weeks.

#### Papain-CPC DDM

In papain-CPC DDM, 2–3 ml of 4% papain-4% CPC working solution [the stock solutions of CPC and papain were prepared by dissolving 4 g of CPC (Himedia, Mumbai, India)] and 4 g of papain (Himedia, Mumbai, India) in 100 ml of distilled water] was added to a 14 ml BD Falcon tube containing 2–3 ml of sputum aliquot. The contents were mixed by vortexing (1 min), and were incubated at 37°C for 30 min. After incubation, the volume was brought to 14 ml with the addition of distilled water. Then, this mixture was thoroughly mixed by inversions and vortexing (1 min) and centrifuged at 3000 x g for 20 min (R-83, Remi, Mumbai, India). The supernatant was carefully decanted and the remaining sediment was re-suspended in 200 μl of distilled water. This re-suspended pellet (0.2 ml) was inoculated on two slants of LJ media (one media with glycerol and the other with sodium pyruvate) followed by incubation at 37°C (aerobically) for 8 weeks aerobically with manual monitoring of once per week for the growth of MTB.

#### Pepsin-CPC DDM

For pepsin-CPC DDM, 2–3 ml of sputum aliquot was mixed with equal quantity (2–3 ml) of 4% pepsin-CPC working solution [the stock solution of pepsin and CPC were prepared by dissolving 4 g of pepsin (Himedia, Mumbai, India) and 4 g of CPC (Himedia, Mumbai, India) in 100 ml of distilled water] and vortexed (1 min). Then, pepsin-CPC method was performed as described in the papain-CPC DDM.

#### Identification of mycobacterial isolates

Growth of MTB on LJ media was confirmed by Ziehl-Neelsen staining, pigment production, growth rate, susceptibility to p-nitrobenzoic acid, niacin utilization test and nitrate reduction test [15]. Performance of each new lot of LJ media were tested using reference strains [M. tuberculosis (ATCC H37Rv) and M. kansasii (ATCC 12478)] and manufactures instructions were strictly followed during the procedure. All isolates of MTB (identified by morphological and biochemical reactions) were further confirmed by a conventional polymerase chain reaction (PCR) using a set of primers designed to amplify an insertion sequence of ‘IS6110’ in the MTB. The sequences of primers used were: T4- 5'-CCT GCG AGC GTA GGC GTC GG 3' and T5-5' CTC GTC CAG CGC CGC TTC GG 3' (GeNei^TM^, Bengaluru, India) and the expected band size was 123 bps [16].

#### Statistical analysis

Diagnostic accuracy was calculated against NALC-NaOH DDM (reference methods) in terms of sensitivity, specificity, positive predictive value, negative predictive value for papain-CPC and pepsin-CPC DDMs. The differences in the sensitivities of the papain-CPC, pepsin-CPC and NALC-NaOH DDMs were compared using McNemar test (performed by GraphPad Prism online version). Fisher’s exact test was used to compare difference in the number of MTB isolates obtained in all the three DDMs investigated.

#### Ethics statement

Maharishi Markandeshwar University ethical committee (MMIMSR/IEC/2014/46) cleared the study protocol. Further, to include in the study, an informed verbal consent was obtained from all the study participants in presence of an independent healthcare worker, who witnessed for voluntary informed decision making of participants.

## Results

**Fig 1:**
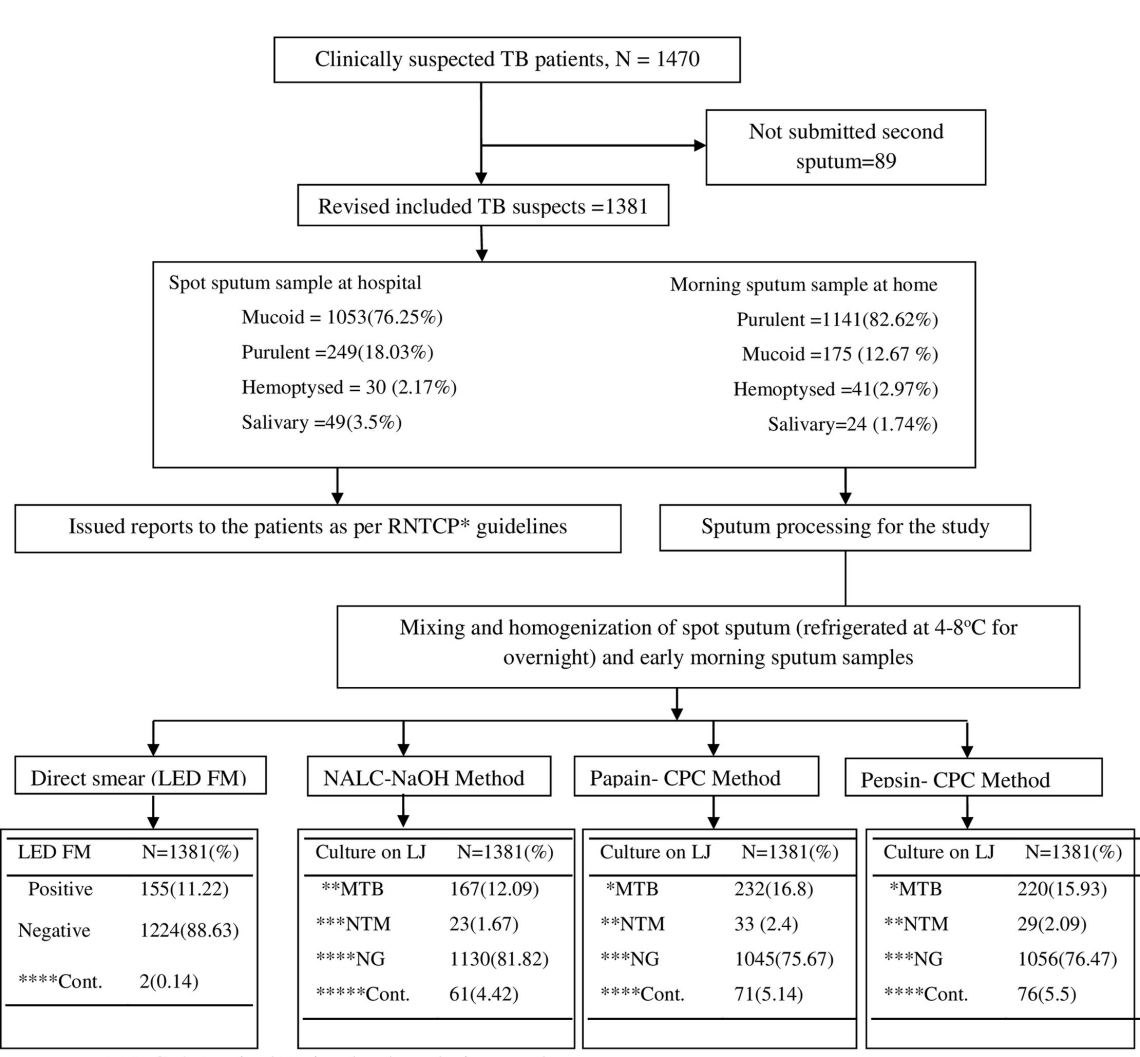
Flow chart showing study profile.

The study profile, test results are depicted in Fig 1 and Table 1, respectively. It was noted that two smear positive sputum samples (smear grade 3+ and 2+) were contaminated in all techniques studied. Further, the difference in the contamination rate of NALC-NaOH and papain-CPC DDM and pepsin-CPC method was insignificant. After excluding 5.5% (76/1381) invalid culture results (obtained in all culture techniques investigated), 12.8% (167/1305), 17.78% (232/1305) and 16.86% (220/1305) patients were confirmed with TB and 1.76% (23/1305), 2.53% (33/1305), 2.22% (29/1305) with nontuberculous mycobacterial (NTM) infections by culture after digestion and decontamination with NALC-NaOH, papain-CPC and pepsin-CPC DDMs, respectively.

**Table 1:** Valid and invalid results.

Results	Direct AFB smear	Culture		
	(N = 1381) (%)	^a^NALC-NaOH DDM (N = 1381) (%)	^b^Papain-CPC DDM (N = 1381) (%)	^c^Pepsin-CPC DDM (N = 1381) (%)
**Valid test results**				
^d^MTB	146(10.57)	167(12)	232(16.79)	220(15.93)
^e^NTM	-	23(1.67)	33(2.4)	29(2)
No growth	9	1130(81.8)	1045(75.67)	1056(76.46)
Subtotal	155(11.22)	1320(95.58)	1310(94.86)	1305(94.5)
**Invalid test results**				
Contaminated cultures				
Bacterial	2(0.14)	59(4.27)	65(4.71)	68(4.92)
Fungal	-	-	1(0.07)	-
^f^Liquefied LJ media	-	2(0.14)	5(0.36)	8(0.57)
Sub total	2(0.14)	61(4.42)	71(5.14)	76(5.5)

^a^N-acetyl-L-cysteine-sodium hydroxide digestion and decontamination method

^b^Papain- *cetylpyridinium chloride d*igestion and decontamination method

^c^Pepsin- *cetylpyridinium chloride* digestion and decontamination method

^d^*Mycobacterium tuberculosis;*

^e^Nontuberculous mycobacteria

^f^No bacterial/fungal elements seen in microscopic examination.

Table 2 summarize culture results obtained after digestion and decontamination with NALC-NaOH, papain-CPC and pepsin-CPC DDM as shown by LED-FM direct microscopy. Of the LED FM smear positive sputum specimens (N = 155), 87.42% (146/167) MTB isolates were obtained when specimens treated with NALC-NaOH DDM (Table 2). However, culture demonstrated growth of MTB in all LED FM smear positive specimens when treated with papain-CPC (N = 232) and pepsin-CPC DDMs (N = 220). Further, it is apparent from Table 2 that the papain-CPC DDM (N = 232) detected twelve additional cases (Fishers exact test, P<0.0005) of MTB when compared to pepsin-CPC DDM (N = 220). Of the total NTM isolates obtained (N = 33), it was noted that papain-CPC DDM (N = 33) detected ten additional cases of NTM (Fishers exact test, P<0.001) when compared to NALC-NaOH DDM (N = 23). Similarly, pepsin-CPC DDM (N = 29) detected six additional cases of NTM (Fishers exact test, P<0.005) in comparison with NALC-NaOH DDM (N = 23). However, when the performance of papain-CPC DDM (N = 33) was compared to pepsin-CPC DDM (N = 29), papain-CPC DDM detected four additional cases of NTM (statistically insignificant). It is evident from Table 2 that of all the MTB isolates obtained after treatment with NALC-NaOH (N = 167), papain-CPC (N = 232) and pepsin-CPC (N = 220) DDMs, 44.31% (74/167), 38.79% (90/232), 33.64% (74/220) of the sputum samples were having higher smear grades (both 3+ and 2+) and demonstrated heavy growth of MTB in culture (3+ and 2+ growth). However, sputum samples with lower smear scores (1+ and scanty) showed relatively less number of MTB colonies on LJ culture irrespective of decontamination techniques used.

**Table 2:** Culture results obtained after digestion and decontamination with NALC-NaOH, papain-CPC and pepsin-CPC DDM as indicated by light emitting diode fluorescent microscope (LED-FM) direct microscopy.

Direct Smear Scores (LED FM)		Culture on LJ media														
		NALC-NaOH DDM					Papain-CPC DDM					Pepsin-CPC DDM				
		^a^3+ (N = 60*)	^b^2+ (N = 48*)	^c^1+ (N = 40*)	^d^Scanty (N = 42*)	NG (N = 1115)	^a^3+ (N = 78*)	^b^2+ (N = 62*)	^c^1+ (N = 59*)	^d^Scanty (N = 66*)	NG (N = 1040)	^a^3+ (N = 68*)	^b^2+ (N = 48*)	^c^1+ (N = 70*)	^d^Scanty (N = 63*)	NG (N = 1056)
3+ (N = 51)		39	7	2	3	-	40	8	2	1	-	40	6	3	2	-
2+(N = 50)		15	13	7	14	1	24	18	5	3	-	17	15	12	6	-
1+(N = 33)		-	-	10	18	5	1	2	8	22	-	-	3	14	16	-
Scanty(N = 21)		-	12	6	-	3	4	10	5	2	-	3	5	8	5	-
Negative (N = 1150)	MTB	5	13	2	1	1106	7	17	23	30	1040	6	11	19	29	1056
	NTM	1	3	13	6		2	7	16	8		2	8	14	5	

^a^3+—more than 200 colonies

^b^2+ - 100 to 200 colonies

^c^1+ - 20 to 99 colonies

^d^ Scanty—less than 20 colonies, NG- No growth

*indicates presence of nontuberculous mycobacteria (NTM)

Table 3 illustrates growth detection time of MTB and NTM for sputum specimens that treated with papain-CPC, pepsin-CPC, NALC-NaOH DDMs. The mean time to detect the tubercle bacilli after digestion and decontamination with papain-CPC, pepsin-CPC DDMs were 35.2 days (SD 9.73) and 36 days (SD 12.7), respectively when compared to the mean detection time [36.07 days (SD 12.63)] of NALC-NaOH DDM (Table 3). It is also obvious from Table 3 that of all the MTB isolates obtained in NALC-NaOH DDM (N = 167), papain-CPC DDM (N = 232) and pepsin-CPC DDM (N = 220), 40.72% (68/167), 37% (86/232) and 35% (77/220) of the sputum samples [having higher smear grades (both 3+ and 2+)] demonstrated growth of tubercle bacilli within 2–4 weeks. However, growth of tubercle bacilli was delayed in majority of the sputum samples with lower smear grades (1+ or scanty) irrespective of the decontamination techniques used. Similarly, the mean time to detection of NTM was found to be 24.93 days (SD 10.59), and 26.62 days (SD 8.23), for papain-CPC and pepsin-CPC DDMs, respectively when compared to the mean detection time of NALC-NaOH DDM [27.04 days (SD 9.68)].

**Table 3:** Growth detection time of Mycobacterium tuberculosis (MTB) and nontuberculous mycobacteria (NTM) for sputum specimens treated with papain-CPC, pepsin-CPC, NALC-NaOH DDMs as indicated by LED-FM smear scores.

Growth detection time (weeks)	LED-FM direct smear scores as per *RNTCP guidelines																	
	NALC-NaOH DDM						Papain-CPC DDM						Pepsin-CPC DDM					
	3+ N = 51	2+ N = 50	1+ N = 33	Scanty N = 21	Negative N = 1150		3+ N = 51	2+ N = 50	1+ N = 33	Scanty N = 21	Negative N = 1150		3+ N = 51	2+ N = 50	1+ N = 33	Scanty N = 21	Negative N = 1150	
					MTB	NTM					MTB	NTM					MTB	NTM
≤1	-	-	-	-	-	2	-	-	-	-	-	3	-	-	-	-		3
2	13	9	1	-	2	3	16	14	3	-	4	4	15	11	3	-	2	2
4	25	21	3	-	1	7	29	27	4	3	9	14	28	23	2	1	11	13
6	9	11	17	6	13	11	4	5	16	7	34	11	6	10	19	7	29	11
8	4	8	7	12	5	-	2	4	10	11	30	1	2	6	9	13	23	-
No growth	-	1	5	3	1106		-	-	-	-	1040		-	-	-	-	1056	

*Revised National Tuberculosis Control Program

Table 4 illustrates the diagnostic accuracy of NALC-NaOH DDM with direct microscopy, papain-CPC and pepsin-CPC DDMs for the detection of MTB and NTM. It is obvious from Table 4 that papain-CPC DDM achieved a higher sensitivity and specificity of 100% and 93.27%, respectively when compared to NALC-NaOH DDM (McNemer test, P<0.0001) for detection of both MTB and NTM. Similarly, pepsin-CPC DDM demonstrated a sensitivity and specificity of 98.94% and 94.71%, respectively as compared to NALC-NaOH DDM (McNemer test, P<0.0001) for detection of both MTB and NTM.

**Table 4:** Diagnostic accuracy of NALC-NaOH DDM with direct microscopy, papain-CPC and pepsin-CPC DDMs for the detection of Mycobacterium tuberculosis (MTB) and nontuberculous mycobacteria (NTM).

Methods	^a^ Culture		Sensitivity (%)	95% C.I.	Specificity (%)	95% C.I.	PPV (%)	95% C.I.	NPV (%)	95% C.I.
Direct LED-FM smear	Positive	Negative								
Positive	146	9	76.8	70.06–82.51	99.19	98.41–99.61	94.19	88.93–97.14	96.17	94.85–97.18
Negative	44*	1106								
Papain–CPC DDM										
Positive	190*	75	100	97.52–100	93.27	91.6–94.64	71.7	65.8–76.96	100	99.54–100
Negative	-	1040								
Pepsin–CPC DDM										
Positive	188*	59	98.94	95.85–99.82	94.71	93.18–95.92	76.11	70.21–81.19	99.81	99.24–99.97
Negative	2*	1056								

^a^ Number of specimens positive (N = 190) and negative (N = 1115) after treatment with NALC-NaOH DDM

*Indicates presence of NTM obtained in culture after treatment with NALC-NaOH (N = 23), papain-CPC (N = 33), pepsin-CPC DDMs (N = 29).

## Discussion

The current study evaluated the performance of NALC-NaOH DDM (reference method) with newly proposed papain-CPC and pepsin-CPC DDMs for the recovery of mycobacteria from sputum specimens that collected from clinically suspected PTB patients. The digested and decontaminated specimens were inoculated on two slants of LJ media; one LJ media with sodium pyruvate (for isolation of *M*. *bovis*) and other with glycerol (for recovery of MTB). However, no *M*. *bovis* strains were isolated in the current study. This may be due to the low prevalence of M. *bovis* in this geographical area^2^. In this study, LJ culture demonstrated growth of MTB in 12.8% of specimens when subjected to NALC-NaOH DDM. These culture results were consistent with previous report wherein the LJ culture detected 10–22.2% of MTB isolates when the sputum specimens cultured after digestion and decontamination with NALC-NaOH DDM [2, 4, 6, and 8]. In the current study, all the MTB isolates (identified by morphological and biochemical reactions), were further confirmed by IS6110 PCR assay. However, this assay couldn’t amplify DNA of five MTB isolates that were identified by conventional methods. This may be probably due to the absence of IS6110 elements in these MTB strains or may also be due to the presence of some PCR inhibitors present. The current study used sputum specimens that were collected at microscopy-designated center of RNTCP (for routine microscopic examination) to presumptively diagnose PTB. However, final diagnosis of PTB was based on direct sputum smear, culture, chest X-ray examination, histopathological evidences and clinical characteristics or as recommended by trained physician. Patients with smear positive cases (and other investigations suggestive of TB) usually started with treatment as per the RNTCP guidelines [17]. However, the physician advises the smear negative patients to submit another sputum sample for culture and Cartridge Based Nucleic Acid Amplification Test after analyzing clinical characteristics and other investigations (standard protocol followed by RNTCP to diagnose PTB) [17]. Therefore, it was less likely to miss a positive case of TB. Moreover, the current study was an experimental design that aimed to evaluate the performance of newly proposed DDMs against NALC-NaOH DDM and therefore, the results of the study were communicated neither with the physicians nor the patients. The other features of the current study design was the use of pooled sputum samples. Indeed; of the two sputum samples (spot sputum and early morning), most of the samples that obtained from same patients were having different consistencies (such as mucoid /salivary/ mucopurulent / blood tinged), insufficient quantity (most of the patients expectorated 4–6 ml of sputum) and variation in the smear scores as well. Therefore, it was essential to obtain a homogeneity in the sputum samples particularly variations in the smear scores. Hence, the sputum samples mixed and homogenized before subjecting to DDMs. Further, it is evident from Table 1 that isolation rates of NTMs were low in all the techniques studied. This was in accordance with earlier studies (from same geographical area) wherein the prevalence of NTM infections varied between 1 to 3.5%, suggesting its clinical relevance [18, 19]. However, Williams and Falkinham et al reported that CPC decontamination might affect the viability of NTM, depending on the species present in the specimens [20]. Further, it was noticed that none of the NTM culture positive sputum specimens could yield positive results in direct AFB smears. This may be attributed to patient inclusion criteria adopted, in fact; most of the patients recruited in the current study might have taken self-medication with macrolides or quinolones in the initial stages of respiratory tract infection (that in turn might have reduced the number of NTM in the sputum to provide a positive smear microscopy).

In general, contamination of culture media is considered to be a major concern in the isolation process of MTB [21]. However, it is evident from Table 1 that the rate of contamination was found to be low for all the methods investigated. These results were also comparable with previous studies conducted wherein the NALC-NaOH and CPC-sodium chloride contamination rate was found to be 2.5% to 3% and 3.5% to 4%, respectively [2, 8]. Further, it is evident from Table 1 that bacterial contamination was predominant in all the methods studied followed by fungal contamination and liquefaction of LJ media. The presence of contamination was ascertained based on the microscopic results obtained in the ZN staining that was performed to confirm growth of MTB in each LJ culture. However, we have not performed any additional tests for further characterization of contaminating bacteria. Further, the fungal growth was identified based on the macroscopic examination, in fact a tube filling growth was observed and that presumptively identified as Zygomycetes species.

LED FM remains the leading diagnostic test for most of the primary health centers and peripheral TB laboratories with limited resources [13]. In the present study, LED FM microscopy could demonstrate a sensitivity and specificity of 76.8% and 99.19%, respectively. This higher sensitivity and specificity of LED FM direct microscopy can be implicated to various factors like patient inclusion criteria, burden of TB, quality of sputum and number of organisms present in sputum [22–25]. In fact, patients having cough for more than two weeks or longer periods were included in this study. However, most of the patients overlook these symptoms (because of lack of awareness of TB and other socioeconomic factors) and may visit hospital after one or two months after primary symptoms. This period must be adequate for TB bacilli to multiply and cause severe pulmonary as well as extra-pulmonary infections. This rationale might perhaps have increased the sensitivity of smear microscopy. However, LED FM failed to detect presence of tubercle bacilli in 1.5%, 5.5% and 4.7% of MTB culture positive specimens that processed using NALC-NaOH, papain-CPC and pepsin-CPC DDMs, respectively. This low sensitivity of direct microscopy may be attributed to the paucibacillary nature of the sputum specimens. Indeed, direct microscopy requires 10000 bacilli per ml of sputum to provide a positive smear microscopy result. In addition, it is evident from Table 3 that LJ culture demonstrated early growth of MTB in majority of the sputum specimens having higher smear grades. This can be explained on the fact that as the smear score increases, the number of bacilli in the specimen also increases, subsequently resulting in reduction of growth detection time.

In the present study, LJ culture demonstrated growth of MTB in smear negative sputum specimens (N = 21) after treatment with NALC-NaOH DDM. This higher recovery rate of MTB in culture (as compared to direct LED FM) is likely due to the increased sensitivity of culture that detects 10–100 bacilli/ml of specimen. Further, NALC-NaOH DDM possesses few limitations such as; technically intricate, instability of NALC solution, and relatively expensive (5g cost ~ 500 INR). In the present study, a diminished colony count was also noted when samples were digested and decontaminated using NALC-NaOH DDM as compared to papain-CPC and pepsin-CPC DDMs. This reduction in number of colonies may be due to death of bacilli in the sputum samples because of the toxicity induced by NaOH during the treatment procedure. In this study, around 20% of the positive cultures were isolated on 8^th^ week of incubation in all the DDMs investigated and this was consistent in other methods as well. Further, there was no significant differences noted in the 8^th^ week of incubation (on the recovery rate of mycobacteria) between all the DDMs studied (Table 3). However, standard length of incubation in many laboratories for reporting culture results is 42 days (6 weeks). This may be because of the fact that most of the laboratories use automated culture systems for the isolation of mycobacteria and that use liquid culture media (considering the fact that mycobacteria multiply well in the liquid media than solid media). However, it is evident from current study that if solid media used for recovery of mycobacteria, the time to detection may be prolonged (>42 days) particularly for specimens having scanty bacilli. This may be because of several factors such as percentage of viability of tubercle bacilli inoculated, type of mycobacterial strain, chemical characteristics of specimens, and the endemicity of the diseases as well.

In the papain-CPC and pepsin CPC DDMs, both papain and pepsin functions as mucolytic agent and CPC as decontaminating agent. In our earlier study, it was found that overnight incubation of sputum with 1% CPC could effectively decontaminate the specimens [2]. However, the concentration of CPC in the current study was increased to 4% to reduce the duration of sputum pretreatment (to be exact; to reduce the overnight incubation of sputum specimens in our previous study to 30 min). This particular concentration (4% papain-4% CPC and 4% pepsin-4% CPC) and duration (30 min) for digestion and decontamination of sputum was achieved from a pilot study conducted using six varying concentrations (briefly; 0.5%, 1%, 2%, 3%, 4% and 5%) of papain-CPC and pepsin-CPC wherein each concentration was tested against ten LED FM smear positive sputum specimens for 30 min. This pilot study concluded that the minimum concentration of 4% papain- 4% CPC and 4% pepsin- 4% CPC DDM could effectively digest and decontaminate the sputum to yield the maximum MTB isolates compared to other concentrations. The other interesting result noted in the current study was the ratio between smear positive and culture positive sputum samples. Of the total culture positive sputum samples obtained after treatment with NALC-NaOH DDM (N = 167), 87.42% (146/167) of the sputum specimens were smear positive. However, the culture positivity of smear positive sputum specimens ranged between 66–70% when the sputum specimens digested and decontaminated using papain-CPC (155/232) and pepsin-CPC DDMs (155/220) (Table 3). This data suggests that most of culture positive sputum samples were paucibacillary. Further, this distinction in the detection rates of mycobacteria (between NALC-NaOH DDM, papain-CPC and pepsin-CPC DDMs) may be attributed to the potential of CPC to preserve the viability of tubercle bacilli much effective than NALC-NaOH DDM. Indeed, it is estimated that NALC-NaOH DDM that uses final specimen concentration of 1% NaOH, even at this low concentration, the loss of tubercle bacilli would be 10^4^ colony-forming units per ml [26]. The other probable reason for this increased sensitivity could be due to the proteolytic activity of both papain and pepsin, which is a protease enzyme that could effectively digest the sputum specimens to release the TB bacilli from the mucus clumps as well as the lysed macrophages present in the sputum specimens. In addition, the papain-CPC and pepsin CPC DDM possess many advantages that include; stability of the solution (can be stored up to 5–7 days if once solubilized), easy to prepare, no sterilization of the solution required, cost-effectiveness (papain and pepsin (500 g each) cost ~500 INR and ~400 INR, respectively) and ready availability. The other advantages of CPC based DDMs include sediments of CPC-treated specimens can be directly inoculated on LJ media as the phospholipids in the egg based media may neutralize the residuals of CPC [8]. However, Ardizzoni et al reported that recovery rate of MTB can be further enhanced when CPC treated specimens were neutralized with buffers containing monopotassium phosphate, sodium thiosulfate and aryl sulfonate [27]. Although, one of the limitations of CPC based DDMs is that CPC treated specimens may have a negative effect when cultured in automated culture systems that uses liquid media [8, 28]. Nevertheless, considering the rate of recovery of tubercle bacilli in CPC based DDMs, future studies are necessary to investigate the effect of neutralizing buffers and its compatibility with liquid media to enhance the recovery rate of mycobacteria from clinical specimens.

## Conclusions

In summary, both papain-CPC and pepsin-CPC DDM could effectively isolate reasonably higher number of mycobacterial isolates particularly in sputum specimens with smear-negative cases as compared to NALC-NaOH DDM. However, when the overall performances of both papain-CPC and pepsin-CPC DDMs compared, papain-CPC DDM could isolate increased number of mycobacterial isolates with comparatively higher number of colonies on LJ media as compared to both pepsin-CPC DDM and NALC-NaOH DDM, indicating the potential of papain-CPC DDM to replace the currently available NALC-NaOH DDM for the recovery of mycobacteria from sputum samples.
